# Supplementary material for: Unveiling the Bioactive Potential of the Invasive Jellyfish Phyllorhiza punctata Through Integrative Transcriptomic and Proteomic Analyses
Source: Biomolecules. 2025 Aug 4;15(8):1121. doi: 10.3390/biom15081121 (PMC12383608; doi:10.3390/biom15081121)
Supplement: Supplementary file 1 [file biomolecules-15-01121-s001.zip › Suplementary Material/FileS3/PEPFOLD4/XP_065066843_13614/clusters.html]

 PEP-FOLD clusters


Download archive of all models  here   

|  |  |  |
| --- | --- | --- |
| # | file | sOPEP |
| # cluster 1 - Size: 26 |  |  |
|  | XP\_065066843\_13614-model1 | -14.2397 |
|  | XP\_065066843\_13614-model1.1 | -14.2394 |
|  | XP\_065066843\_13614-model1.2 | -14.2394 |
|  | XP\_065066843\_13614-model1.3 | -14.2394 |
|  | XP\_065066843\_13614-model1.4 | -14.2271 |
|  | XP\_065066843\_13614-model1.5 | -14.2037 |
|  | XP\_065066843\_13614-model1.6 | -14.2037 |
|  | XP\_065066843\_13614-model1.7 | -14.2037 |
|  | XP\_065066843\_13614-model1.8 | -14.2037 |
|  | XP\_065066843\_13614-model1.9 | -14.1989 |
|  | XP\_065066843\_13614-model1.10 | -14.1989 |
|  | XP\_065066843\_13614-model1.11 | -14.1989 |
|  | XP\_065066843\_13614-model1.12 | -14.1989 |
|  | XP\_065066843\_13614-model1.13 | -14.1989 |
|  | XP\_065066843\_13614-model1.14 | -14.175 |
|  | XP\_065066843\_13614-model1.15 | -14.175 |
|  | XP\_065066843\_13614-model1.16 | -14.1741 |
|  | XP\_065066843\_13614-model1.17 | -14.1741 |
|  | XP\_065066843\_13614-model1.18 | -14.1741 |
|  | XP\_065066843\_13614-model1.19 | -14.1487 |
|  | XP\_065066843\_13614-model1.20 | -14.1487 |
|  | XP\_065066843\_13614-model1.21 | -14.1276 |
|  | XP\_065066843\_13614-model1.22 | -14.103 |
|  | XP\_065066843\_13614-model1.23 | -14.103 |
|  | XP\_065066843\_13614-model1.24 | -14.0903 |
|  | XP\_065066843\_13614-model1.25 | -14.079 |
| # cluster 2 - Size: 35 |  |  |
|  | XP\_065066843\_13614-model2 | -14.231 |
|  | XP\_065066843\_13614-model2.1 | -14.194 |
|  | XP\_065066843\_13614-model2.2 | -14.1905 |
|  | XP\_065066843\_13614-model2.3 | -14.1905 |
|  | XP\_065066843\_13614-model2.4 | -14.1905 |
|  | XP\_065066843\_13614-model2.5 | -14.1883 |
|  | XP\_065066843\_13614-model2.6 | -14.1883 |
|  | XP\_065066843\_13614-model2.7 | -14.1883 |
|  | XP\_065066843\_13614-model2.8 | -14.1883 |
|  | XP\_065066843\_13614-model2.9 | -14.1883 |
|  | XP\_065066843\_13614-model2.10 | -14.1883 |
|  | XP\_065066843\_13614-model2.11 | -14.1883 |
|  | XP\_065066843\_13614-model2.12 | -14.1883 |
|  | XP\_065066843\_13614-model2.13 | -14.1883 |
|  | XP\_065066843\_13614-model2.14 | -14.1883 |
|  | XP\_065066843\_13614-model2.15 | -14.1768 |
|  | XP\_065066843\_13614-model2.16 | -14.1768 |
|  | XP\_065066843\_13614-model2.17 | -14.1723 |
|  | XP\_065066843\_13614-model2.18 | -14.1688 |
|  | XP\_065066843\_13614-model2.19 | -14.152 |
|  | XP\_065066843\_13614-model2.20 | -14.1513 |
|  | XP\_065066843\_13614-model2.21 | -14.1513 |
|  | XP\_065066843\_13614-model2.22 | -14.1513 |
|  | XP\_065066843\_13614-model2.23 | -14.1513 |
|  | XP\_065066843\_13614-model2.24 | -14.1513 |
|  | XP\_065066843\_13614-model2.25 | -14.1513 |
|  | XP\_065066843\_13614-model2.26 | -14.1513 |
|  | XP\_065066843\_13614-model2.27 | -14.1513 |
|  | XP\_065066843\_13614-model2.28 | -14.1201 |
|  | XP\_065066843\_13614-model2.29 | -14.1147 |
|  | XP\_065066843\_13614-model2.30 | -14.1147 |
|  | XP\_065066843\_13614-model2.31 | -14.1007 |
|  | XP\_065066843\_13614-model2.32 | -14.0821 |
|  | XP\_065066843\_13614-model2.33 | -14.079 |
|  | XP\_065066843\_13614-model2.34 | -14.0515 |
| # cluster 3 - Size: 10 |  |  |
|  | XP\_065066843\_13614-model3 | -14.2258 |
|  | XP\_065066843\_13614-model3.1 | -14.2117 |
|  | XP\_065066843\_13614-model3.2 | -14.2117 |
|  | XP\_065066843\_13614-model3.3 | -14.2117 |
|  | XP\_065066843\_13614-model3.4 | -14.2117 |
|  | XP\_065066843\_13614-model3.5 | -14.2117 |
|  | XP\_065066843\_13614-model3.6 | -14.1673 |
|  | XP\_065066843\_13614-model3.7 | -14.1519 |
|  | XP\_065066843\_13614-model3.8 | -14.1519 |
|  | XP\_065066843\_13614-model3.9 | -14.0754 |
| # cluster 4 - Size: 13 |  |  |
|  | XP\_065066843\_13614-model4 | -14.2219 |
|  | XP\_065066843\_13614-model4.1 | -14.2219 |
|  | XP\_065066843\_13614-model4.2 | -14.2219 |
|  | XP\_065066843\_13614-model4.3 | -14.1709 |
|  | XP\_065066843\_13614-model4.4 | -14.1709 |
|  | XP\_065066843\_13614-model4.5 | -14.1709 |
|  | XP\_065066843\_13614-model4.6 | -14.1709 |
|  | XP\_065066843\_13614-model4.7 | -14.1562 |
|  | XP\_065066843\_13614-model4.8 | -14.1562 |
|  | XP\_065066843\_13614-model4.9 | -14.1301 |
|  | XP\_065066843\_13614-model4.10 | -14.0967 |
|  | XP\_065066843\_13614-model4.11 | -14.0854 |
|  | XP\_065066843\_13614-model4.12 | -14.07 |
| # cluster 5 - Size: 15 |  |  |
|  | XP\_065066843\_13614-model5 | -14.2096 |
|  | XP\_065066843\_13614-model5.1 | -14.2096 |
|  | XP\_065066843\_13614-model5.2 | -14.2096 |
|  | XP\_065066843\_13614-model5.3 | -14.1866 |
|  | XP\_065066843\_13614-model5.4 | -14.1866 |
|  | XP\_065066843\_13614-model5.5 | -14.1371 |
|  | XP\_065066843\_13614-model5.6 | -14.1316 |
|  | XP\_065066843\_13614-model5.7 | -14.1316 |
|  | XP\_065066843\_13614-model5.8 | -14.1316 |
|  | XP\_065066843\_13614-model5.9 | -14.1176 |
|  | XP\_065066843\_13614-model5.10 | -14.1099 |
|  | XP\_065066843\_13614-model5.11 | -14.1099 |
|  | XP\_065066843\_13614-model5.12 | -14.0634 |
|  | XP\_065066843\_13614-model5.13 | -14.0474 |
|  | XP\_065066843\_13614-model5.14 | -14.0255 |
| # cluster 6 - Size: 1 |  |  |
|  | XP\_065066843\_13614-model6 | -14.0311 |
|  |  |  |
